# Whole-Genome Sequencing Analysis of Quorum Quenching Bacterial Strain *Acinetobacter lactucae* QL-1 Identifies the FadY Enzyme for Degradation of the Diffusible Signal Factor

**DOI:** 10.3390/ijms21186729

**Published:** 2020-09-14

**Authors:** Tian Ye, Tian Zhou, Xudan Xu, Wenping Zhang, Xinghui Fan, Sandhya Mishra, Lianhui Zhang, Xiaofan Zhou, Shaohua Chen

**Affiliations:** 1 State Key Laboratory for Conservation and Utilization of Subtropical Agro-Bioresources, Guangdong Province Key Laboratory of Microbial Signals and Disease Control, Integrative Microbiology Research Centre, South China Agricultural University, Guangzhou 510642, China; 20182047012@stu.scau.edu.cn (T.Y.); 20171021009@stu.scau.edu.cn (T.Z.); xuxudan@stu.scau.edu.cn (X.X.); 20191047008@stu.scau.edu.cn (W.Z.); fxhscau@163.com (X.F.); sandhyamanshi@gmail.com (S.M.); lhzhang01@scau.edu.cn (L.Z.); 2 Guangdong Laboratory for Lingnan Modern Agriculture, Guangzhou 510642, China

**Keywords:** diffusible signal factor, *Acinetobacter lactucae*, whole-genome sequencing, degradation enzyme, biocontrol, *Xanthomonas campestris* pv. *campestris*

## Abstract

The diffusible signal factor (DSF) is a fatty acid signal molecule and is widely conserved in various Gram-negative bacteria. DSF is involved in the regulation of pathogenic virulence in many bacterial pathogens, including *Xanthomonas campestris* pv. *campestris* (*Xcc*). Quorum quenching (QQ) is a potential approach for preventing and controlling DSF-mediated bacterial infections by the degradation of the DSF signal. *Acinetobacter lactucae* strain QL-1 possesses a superb DSF degradation ability and effectively attenuates *Xcc* virulence through QQ. However, the QQ mechanisms in strain QL-1 are still unknown. In the present study, whole-genome sequencing and comparative genomics analysis were conducted to identify the molecular mechanisms of QQ in strain QL-1. We found that the *fadY* gene of QL-1 is an ortholog of *Xcc* *rpfB*, a known DSF degradation gene, suggesting that strain QL-1 is capable of inactivating DSF by QQ enzymes. The results of site-directed mutagenesis indicated that *fadY* is required for strain QL-1 to degrade DSF. The determination of FadY activity in vitro revealed that the fatty acyl-CoA synthetase FadY had remarkable catalytic activity. Furthermore, the expression of *fadY* in transformed *Xcc* strain XC1 was investigated and shown to significantly attenuate bacterial pathogenicity on host plants, such as Chinese cabbage and radish. This is the first report demonstrating a DSF degradation enzyme from *A. lactucae*. Taken together, these findings shed light on the QQ mechanisms of *A. lactucae* strain QL-1, and provide useful enzymes and related genes for the biocontrol of infectious diseases caused by DSF-dependent bacterial pathogens.

## 1. Introduction

*Xanthomonas campestris* pv. *campestris* (*Xcc*) a causal agent of black rot, the most important and harmful plant disease known to cruciferous plants. It can infect all cultivable varieties of *Brassica* vegetables, crops, and ornamental and weed plants worldwide [1]. Upon infection of the host plant, *Xcc* produces a range of extracellular enzymes, which collectively play crucial roles in pathogenesis. The quorum sensing (QS) mechanism is mainly responsible for the production of these factors, mediated by the diffusible signal factor (DSF) signaling molecule. *Xcc* expresses tissue-macerating pathogenicity genes upon the accumulation of DSF, and has been characterized as *cis*-11-methyl-2-dodecenoic acid [2,3]. DSF represents a family of widely conserved QS signals involved in the regulation of virulence factor production in a variety of Gram-negative bacteria. DSF not only exists in all *Xanthomonas* sp., but also widely exists in a variety of *Burkholderia* sp., *Pseudomonas aeruginosa*, and marine bacteria [4]. For the treatment and control of black rot caused by *Xcc*, chemical pesticides and antibiotics are largely used, leading to critical environmental pollution; destruction of the ecological balance; and a series of serious problems, such as food safety. In addition, the overproduction and overuse of pesticides and antibiotics have caused more and more pathogenic bacteria to develop drug resistance, and even multiple drug resistance, problems [5,6,7]. Therefore, there is a need to find a novel and effective prevention strategy for the control of plant diseases caused by pathogens.

Regarding DSF, quorum quenching (QQ) is an efficient disease prevention and control method for plant diseases that disrupt QS by either the degradation of QS signals or interference of signal generation. QQ means that the signal molecules of the pathogens are quenched to prevent the effective accumulation of signal molecules. When the concentration of the signal molecules is reduced, the expression of pathogenic genes of the pathogen cannot be activated, to destroy the communication between cells and the QS system [8]. The increasing number of microorganisms and QQ enzymes reported hindering quorum sensing supports new perspectives for the development of biocontrol strategies [9].

Research on the application of QQ enzymes focuses on the following four aspects: (1) Transplanting the QQ enzyme gene into plants to obtain transgenic plants. Dong et al. [10] transferred the AiiA lactase gene of Bacillus sp. 240B1 into potato and tobacco to control infection and alleviate the symptoms of plant soft rot, which, for the first time, proved that transgenic plants can degrade the QS signal molecule *N*-acyl-homoserine lactone (AHL) through the expression of its QQ enzyme and block the communication between plant pathogen cells to prevent disease. Subsequent research on transferring the AiiA esterase gene into konjac could significantly weaken the potato black shank disease pathogen (*Pectobacterium carotovorum*) pathogenic [11]; (2) transplanting the QQ enzyme gene into microorganisms to obtain transgenic QQ microorganisms. A research study transplanted the AiiA lactase gene into *Burkholderia* sp. KJ006 to degrade QS signals, which could alleviate rice seedling disease caused by *Burkholderia glumae* [12]. *Bacillus thuringiensis* containing the AiiA lactase gene was proved to be resistant to soft rot in the potato tuber model infection system test [13]; (3) screening QQ microorganisms from the natural environment as biological degradation agents [14,15]. Nhan et al. [16] added a culture of AHL-degrading bacteria from the gastrointestinal tract of bass to the feeding pond of *Macrobrachium rosenbergii* larvae or to the food of shrimp larvae, which could increase the survival rate of shrimp larvae against *Vibrio harveyi*. Defoirdt et al. [17] further demonstrated that Bacillus isolated from a culture of AHL-degrading bacteria is expected to be a probiotic for the prevention and control of animal and plant diseases. Recently, Torres et al. [18] reported a strain of Alteromonas stellipolaris, which could significantly reduce the degree of tissue damage caused by pathogenic *Vibrio*; (4) purified QQ enzymes are directly used to control human and animal pathogens. Studies have shown that purified AHL-degrading enzymes, such as acyltransferase AhlM and PvdQ, can reduce the expression of virulence factors of *Pseudomonas aeruginosa* [19]. The simultaneous injection of AiiA into carp or zebrafish with the pathogenic bacterium *Aeromonas hydrophila* can also reduce infection [20].

The application of AHL-degrading bacteria and their degrading enzymes has been widely investigated in recent years. However, information about DSF-degrading bacteria and their degrading enzymes is rare. To date, genome sequences of DSF-degrading bacterial strains and the molecular mechanisms are still unknown, which limits the further use of these QQ candidates. Therefore, in the present study, we screened for DSF degradation enzymes through whole-genome sequencing (WGS) and comparative genomics analysis of *Acinetobacter lactucae* QL-1, a DSF-degrading bacterial strain previously isolated from an agricultural field [21], and further determined their potential for DSF degradation and biocontrol of the infectious diseases caused by DSF-dependent bacterial pathogens.

## 2. Results

### 2.1. Sequencing and Analysis of the A. lactucae QL-1 Genome

**Figure 1:**
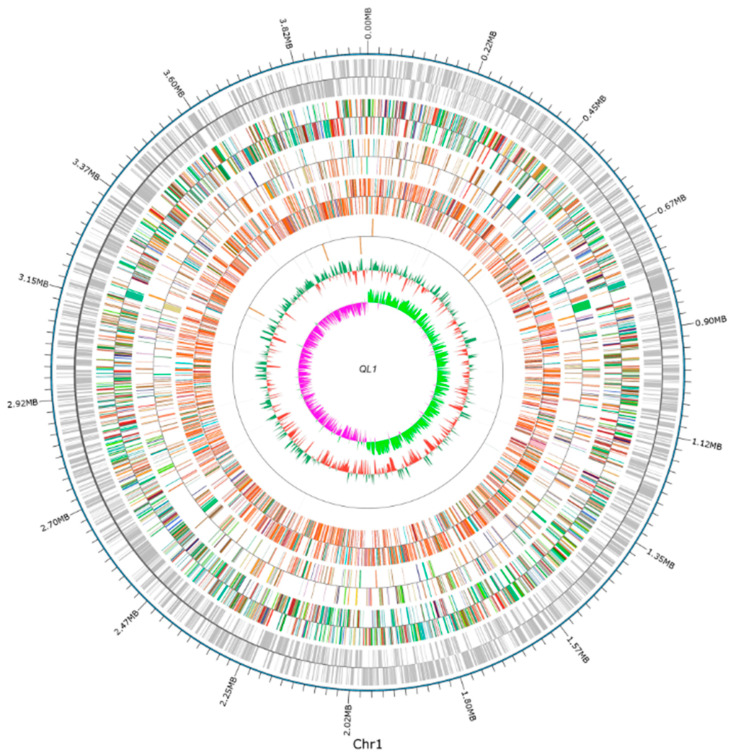
A circular genome diagram of *Acinetobacter lactucae* QL-1 (red: less than mean; green: greater than mean; and the higher the peak, the greater the difference between the mean) and GC skew (GC skew = (G − C)/(G + C); inward pink: G > C, outward light green: G < C), could be fully demonstrated.

The genome of *A. lactucae* QL-1 were sequenced by using both Illumina and PacBio technologies, generating 4,392,245 short-read pairs (1.318 Gb) and 97,120 long reads (0.757 Gb), respectively. The genome characteristics of QL-1 are shown in Table 1. The genome sequencing data were assembled into a complete, circular chromosome (Figure 1), which is 3,973,648 bp in size and has a GC content of 40.4%. The genome assembly has a BUSCO completeness of 99.7% (780 out of 782 genes in the Pseudomonadales data set), indicative of very high quality. A total of 3.707 protein-coding, 73 tRNA, and 18 rRNA genes, as well as 153 repeat elements, were annotated in the QL-1 genome. The total length of protein-coding genes is 3,485,109 bp, accounting for 87.71% of the genome length. Detailed results of the functional annotation of these protein-coding genes and the prediction of other genomic features are presented in the supplementary text.

**Figure 2:**
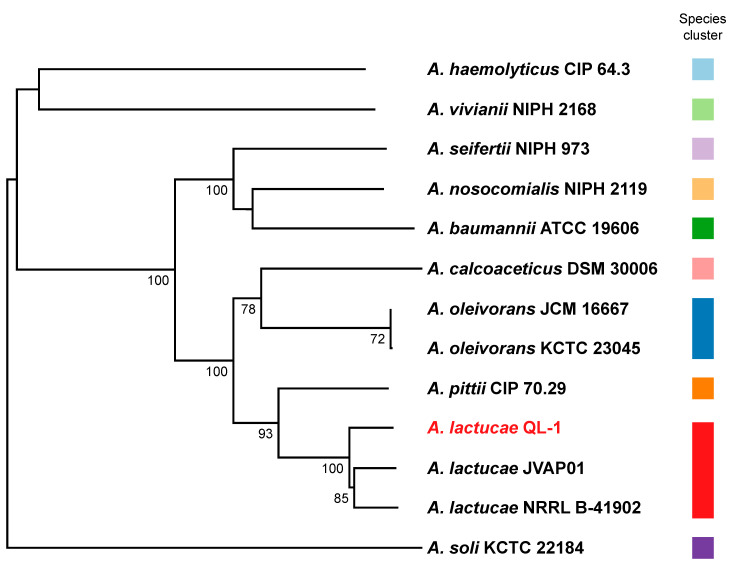
Phylogenetic tee inferred from 13 Acinetobacter genome sequences. The branch lengths are scaled in terms of Genome BLAST Distance Phylogeny (GBDP) distance formula *d*_5_. The numbers above branches are GBDP pseudo-bootstrap support values > 60% from 100 replications, with average branch support of 82.5%. The tree was rooted at the midpoint.

Previously we have classified QL-1 as a strain of *A. lactucae* based on the phylogenetic analysis of 16S rDNA gene sequences. To verify this classification, we performed a genome-based taxonomic analysis of QL-1, and the result confirmed that QL-1 belongs to the species *A. lactucae*. In the genome-based phylogeny of *Acinetobacter*, QL-1, *A. lactucae* NRRL B-41902 (the type strain of *A. lactucae*), and *A. lactucae* JVAP01 formed a monophyletic clade with maximum support and were grouped into the same species cluster (Figure 2). Furthermore, the average nucleotide identity between QL-1 and NRRL B-41092 is 97%, which is above the suggested threshold of 95% for prokaryotic species delimitation [22].

### 2.2. Identification and Cloning of the Gene Responsible for the Degradation of DSF

In *Xcc*, the regulator of the pathogenicity factor (rpf) gene cluster encodes multiple proteins that produce and sense a fatty acid signal molecule called DSF. The *rpf*B gene encodes an acyl-CoA ligase and is involved in DSF degradation [23]. To identify genes responsible for the degradation of DSF in QL-1, we first used InParanoid to delineate orthologs between *A. lactucae* QL-1 and *Xcc*. As a result, we found that QL-1 has two co-orthologs of *Xcc rpfB*, namely, *fadY* and *fadZ*, both of which were predicted to encode long-chain acyl-CoA synthetase. Pfam analysis of FadY, FadZ, and RpfB showed that they all have the same protein domain architectures (PF00501, AMP-binding enzyme; and PF13193, AMP-binding enzyme C-terminal domain). Moreover, all three proteins were annotated with the same KEGG Orthology term K01897, corresponding to long-chain acyl-CoA synthetase (EC:6.2.1.3). Notably, FadY and FadZ are the only two proteins in QL-1 that are assigned with K01897 and EC:6.2.1.3. We then performed a pairwise sequence comparison between RpfB and each of FadY and FadZ. The results showed that RpfB and FadY share a sequence identify of 52% and a similarity of 68%, whereas RpfB and FadZ showed slightly lower identity (48%) and similarity (62%). In fact, RpfB and FadY are reciprocal best hits in the genome-wide comparison between *A. lactucae* QL-1 and *Xcc*. Therefore, we elected to focus our functional investigation on *fadY*, which, comparing to *fadZ*, is more likely to have similar functions as *rpfB*.

**Figure 3:**
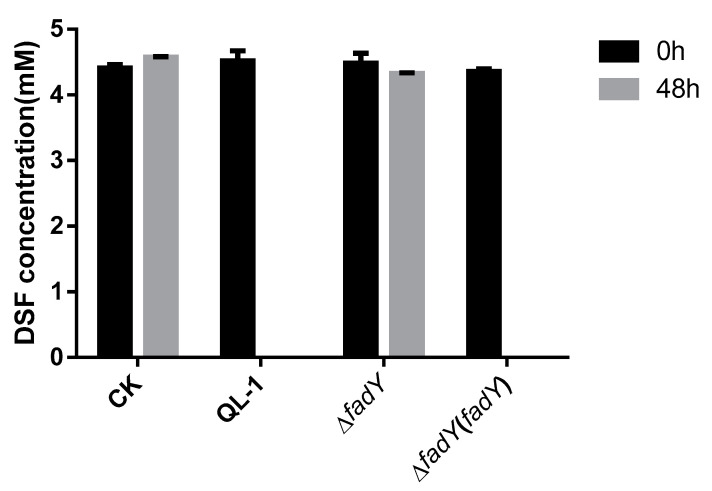
Diffusible signal factor (DSF)-degrading activity comparison of mutants and wild-type strain QL-1. CK: control; strain QL-1: wild-type; ∆*fadY*: *fadY* deletion mutant; ∆*fadY* (*fadY*): the complement of the *fadY* deletion mutant.

To verify whether the FadY enzyme can degrade DSF, we used the pGEX-6p-1 plasmid to express FadY, as described above (Figure S2). To investigate whether *fadY* affects the degradation of DSF, the *fadY* in-frame ∆*fadY* deletion mutant was generated by using strain QL-1 as the parental strain, as described previously. The QL-1 strain and ∆*fadY* were cultured in the MSM medium, amended with DSF as the sole carbon source. As expected, ∆*fadY* could not grow, but strain QL-1 could grow well in the medium. The remaining DSF production in the medium was tested at 0 and 48 h, respectively, to further explore whether strain QL-1 and ∆*fadY* utilized DSF. The comparative study of DSF-degrading activity between the control (CK), wild-type (strain QL-1), and ∆*fadY* cultured in DSF medium for 48 h is shown in Figure 3. We determined that *fadY* is the key gene that influences whether the wild QL-1 can degrade DSF. These results indicated that ∆*fadY* could not utilize DSF as a carbon source in the medium, which further suggested that *fadY* played an important role in the degradation of DSF. For further verification, the wild-type *fadY* was cloned under the control of the *lac* promoter in the plasmid vector pBBR1-MCS-5-generated ∆*fadY* (*fadY*) in *trans* complementation analysis. As expected, ∆*fadY* (*fadY*) regained the ability to degrade DSF (Figure 3).

### 2.3. Activity Detection of FadY

**Figure 4:**
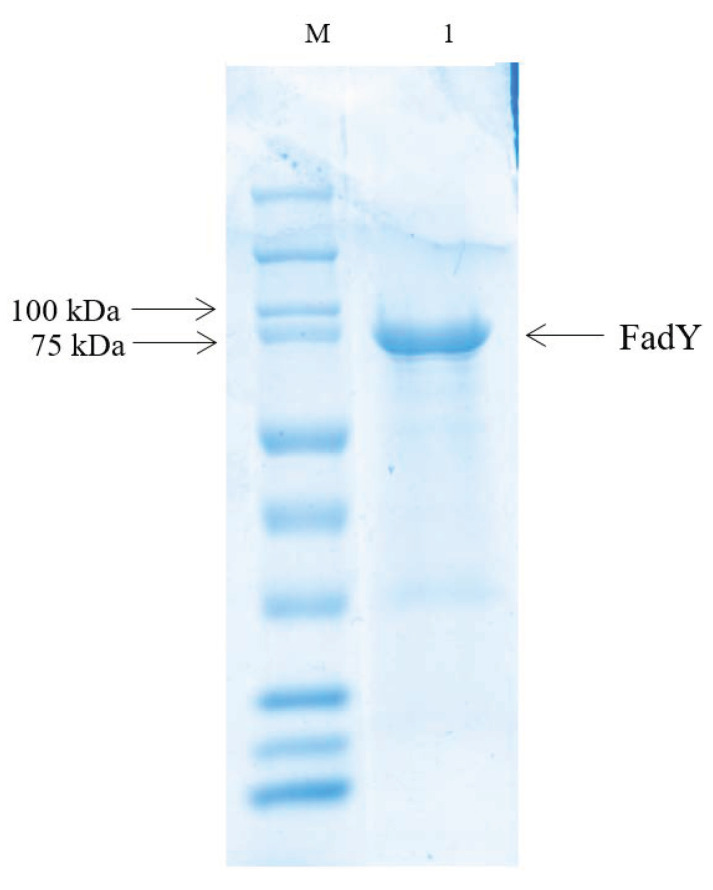
SDS-PAGE analysis of purified FadY. M: Marker; 1: purified FadY.

To perform the following experiment, as described above, an optimized DNA fragment was cloned into plasmid PGEX-6p-1 to express the purity protein (Figure 4). If COASH and FFA reacted with FadY to generate FA-CoA, then, due to the consumption of COASH, DTNB reacted with residual COASH in a reaction to produce a compound that had strong light absorption at 412 nm. The progress of the reaction was reflected by measuring the absorbance of reaction liquid extracted at different stages. The color contrast of the reaction with different treatments is shown in Figure S3.

**Figure 5:**
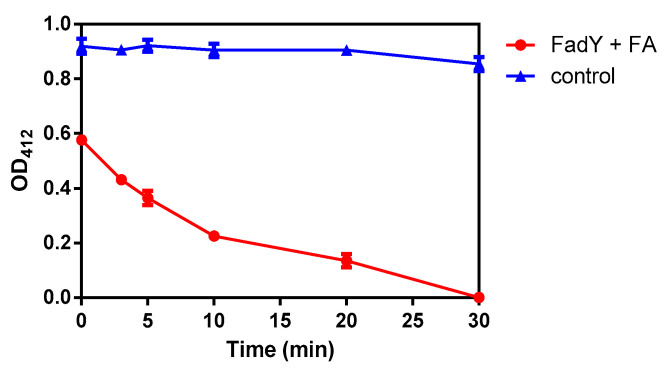
The curve of absorbance versus time in the activity detection of FadY.

The results showed that as the reaction went on, the consumption of COASH increased, which demonstrated that the change in absorbance also increased. According to Table 2, the average absorbance of the mixture (FadY + FA) was 0.576, 0.431 0.364, 0.225, 0.135, and 0 after 0, 3, 5, 10, 20, and 30 min, respectively. The absorbance of the control did not change as the reaction progressed, which verified the stability of CoASH and DTNB under the conditions presented above. Additionally, the average absorbance of the mixture (FadY + FA) measured at the initial stage of the reaction was 0.576, while that of the control was 0.919, indicating that the reaction occurred very quickly. Moreover, after 30 min, the absorbance of the mixture (FadY + FA) was 0, which meant that the substrate had been exhausted, suggesting that FadY was fatty acyl-CoA synthetase with excellent catalytic activity to COASH and sodium oleate. The curve in Figure 5 showed that the absorbance became smaller and smaller as the reaction time progressed. All of the results above revealed that the fatty acyl-CoA synthetase FadY had an excellent activity to be used to carry out the next step.

### 2.4. Xcc Expressing FadY Loses Virulence in Planta

**Figure 6:**
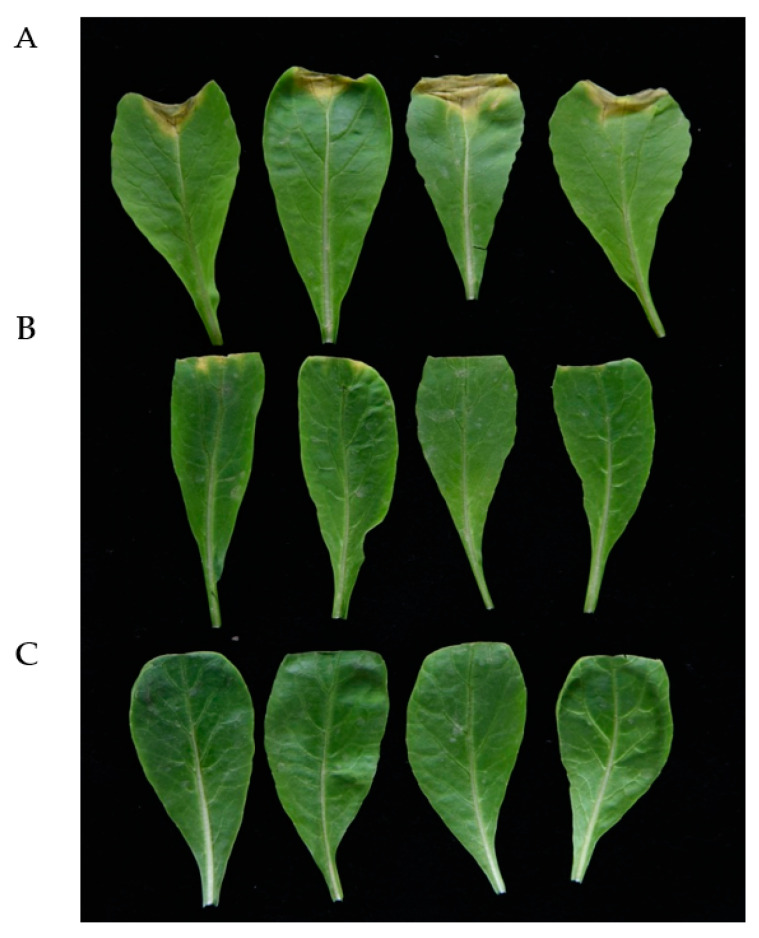
Effect of *fadY* gene expression on *Xcc* pathogenicity. From top to bottom, radish inoculated, respectively, with *Xcc* inoculum (**A**), *Xcc* (FadY) (**B**), and distilled water (**C**). The photographs were taken 30 days after inoculation.

**Figure 7:**
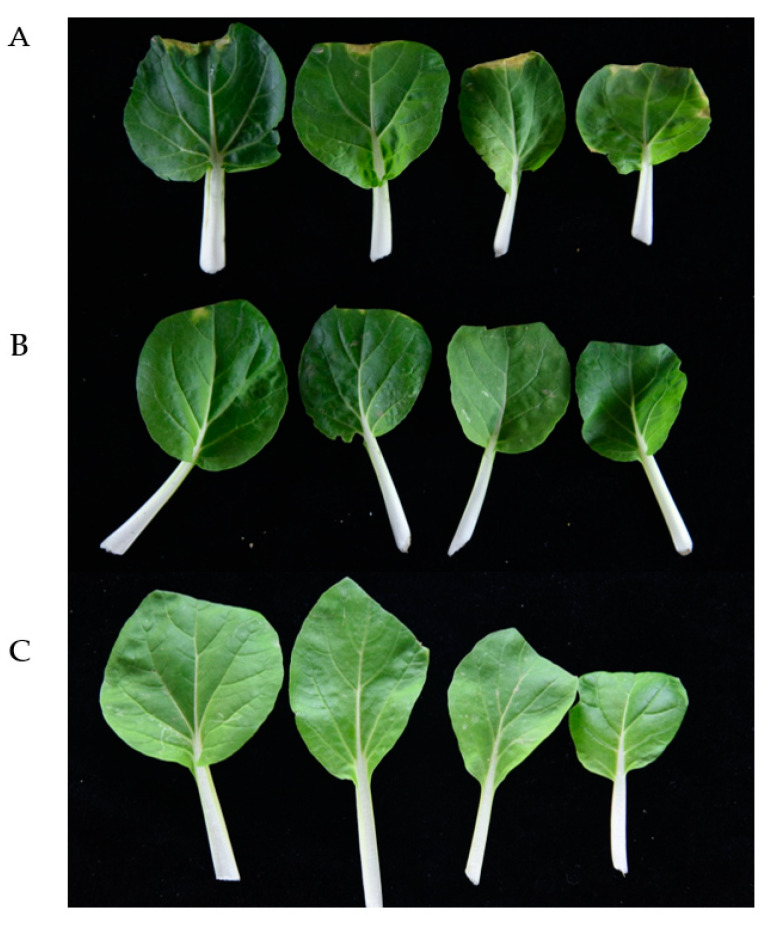
Effect of *fadY* gene expression on *Xcc* pathogenicity. From top to bottom, Chinese cabbage inoculated, respectively, with *Xcc* inoculum (**A**), *Xcc* (FadY) (**B**), and distilled water (**C**). The photographs were taken 30 days after inoculation.

An *Xcc* propagate in young stems and leaves through the vascular system, and disease appears as V-shaped chlorotic to necrotic lesions on leaf margins [24]. To determine the effect of the infection time on symptom development, the bacterial strains were inoculated into intact plants of Chinese cabbage and radish. Black rot symptoms appeared after 10 days of inoculation with wild-type strain *Xcc* and strain *Xcc* (FadY). No significant symptoms or only minor black rot disease symptoms were detected in plants inoculated with the strain a month after inoculation. Figure 6 and Figure 7 show inoculated Chinese cabbages and radish after 30 days of inoculation. *Xcc* that expressed FadY failed to cause black rot disease symptoms in radish and Chinese cabbage as the control (Figure 6 and Figure 7C) or caused only minor black rot disease symptoms (Figure 6 and Figure 7B), whereas its parental strain *Xcc* XC1 caused severe V-shaped lesions on leaf margins (Figure 6 and Figure 7A).

## 3. Discussion

Whole-genome sequencing (WGS) has allowed us to predict the gene structure and function of several unknown bacterial strains. Understanding the diversity, environmental adaptability, and production of various bioactive substances from bacterial strains is of great significance for the control of diseases and other related research in the future [25]. In this study, the complete genome sequence of a quorum quenching bacterial strain *A. lactucae* QL-1 was sequenced and analyzed in order to elucidate the DSF-degrading mechanisms underlying its QQ capability. As a result, the gene (*fadY*), which likely encodes a long-chain acyl-CoA synthetase, has been identified as the ortholog of *rpfB*, a known DSF inactivation enzyme encoding gene of *Xcc*. Further cloning and site-directed mutagenesis of *fadY* was performed, and the DSF-inactivation activity of the mutants and wild-type strain QL-1 was assayed, which confirmed that the gene *fadY* was necessary for the DSF degradation of QL-1. Further studies could focus on the *A. lactucae* QL-1 genes related to DSF metabolite biosynthesis, particularly genes associated with the degradation pathway. The genomic sequence information obtained in this study will provide valuable resources for further efforts to promote the supplementary application of DSF-degrading bacteria or their degrading enzymes. QS is one of the most important discoveries in the field of microbiology in recent years, and confirms that individual microorganisms can exchange their extensive information by generating and sensing small molecular chemical signals, to make a coordinated response to the host and external environment in the form of a population, including establishing infection and producing antibiotics [26,27]. QS provides a new breakthrough point for the prevention and control of plant diseases. The QQ strategy prevents the signal molecules of the pathogen from effectively accumulating through quenching signal molecules; after which, the concentration of signal molecules to reduce cannot activate the expression of pathogen virulence genes, which damage cell communication, undermining the QQ. Many microorganisms, such as *Actinobacteria* sp., *Bacteroidetes* sp., *Firmicutes* sp., and *Proteobacteria* sp., can degrade AHL signal molecules [9,17,18,28,29,30,31]. A number of bacterial strains, capable of degrading DSF have also been identified and characterized, including *Bacillus*, *Paenibacillus*, *Microbacterium*, *Staphylococcus*, *Cupriavidus*, *Acinetobacter*, and *Pseudomonas* [2,21,32,33]. Since the first isolation of AHL lactase AiiA was reported in *Bacillus cereus* [10], and the isolation of AHL amidase in *Variovorax paradoxus*, more and more reports on AHL-degrading enzymes have been successively reported [11,12,13,17,34,35]. However, little is known about DSF-degrading enzymes and genome sequences of DSF-degrading strains. In this study, WGS, comparative genomics analysis, genome component prediction, and the gene function were used to identify the QQ mechanism in *A. lactucae* strain QL-1. This is the first report demonstrating a DSF degradation enzyme from *A. lactucae.*

Because DSF was hard to dissolve in water, sodium oleate, which had a high solubility, was chosen as the substrate replacing DSF for activity detection. Additionally, oleic acid is a kind of FFA that widely exists in nature and accounts for about 54% of the FFA content in human blood, and the other two FFA with higher contents are palmitic acid (about 34%) and stearic acid (about 6%), whose structures are similar to oleic acid [36,37,38,39]. According to the reaction results, FadY exhibited remarkable activity in relation to sodium oleate and COASH.

*Xcc*, as a plant pathogen, produces DSF that act as virulence determinants for black rot diseases of various plants, including *Brassica*, mustard, and canola vegetables [40,41]. It was found that the expression of the AiiA enzyme in *Erwinia carotovora* significantly reduced the release of autoinducers (AIs), decreased extracellular pectolytic enzyme activities, and attenuated soft rot disease symptoms in potato, Chinese cabbage, carrot, and so on [42]. To determine the impact of the *fadY* gene on virulence, a cosmid clone containing the *fadY* gene was introduced into *Xcc* strain XC1. Our results indicated that the expression of the *fadY* enzyme in *Xcc* significantly attenuated black rot disease symptoms in radish and Chinese cabbage. These results further support the potential of the *fadY* gene to confer resistance to black rot disease and other diseases in plants in which DSF is involved in the regulation of pathogenic gene expression.

DSF exists in many Gram-negative bacteria, and some of them are important human pathogens, such as *Burkholderia* sp. and *Pseudomonas aeruginosa* [4,43]. Moreover, other DSF signals have been identified and reported, such as *cis*-2-dodecenoic acid (BDSF) and (2Z,3Z)-11-methyldodeca-2,5- dienoic acid (CDSF), forming the DSF family [4,44,45,46]. Therefore, the *fadY* gene could be a potential tool for investigating the role of the DSF-related regulation of pathogenic gene expression in bacteria. It is feasible and simple to introduce the *fadY* gene into these bacteria to probe the biological functions regulated by DSF.

## 4. Materials and Methods

### 4.1. Strains, Plasmids, and Media

The strains and plasmids used in this study are listed in Table 3. Among them, pGEX-6P-1, the bacterial vector for expressing GST fusion proteins with a PreScission protease site. The expression of this plasmid with the GST label enhances the solubility of the target protein and is widely used in the expression of the target protein, which has not been mentioned the ability of plasmid in killing the bacterial target in a large number of reported literature [24,47,48,49,50]. The screening of bacterial isolate QL-1 (*A. lactucae)* capable of degrading DSF has been described previously [21]. *Escherichia coli* strain DH5*α* was cultured in Luria-Bertani (LB) medium at 37 °C, strain QL-1 and its derivatives were cultured in LB medium at 30 °C, and *Xcc* strain XC1 and its derivatives were cultured in LB at 28 °C. Appropriate antibiotics were added to the media at their respective concentrations: Ampicillin, 100 μg/mL; rifampicin, 50 μg/mL; gentamicin, 50 μg/mL; kanamycin, 50 μg/mL; and tetracycline, 15 μg/mL.

### 4.2. Whole-Genome Sequencing and Assembly

Genomic information helps in gaining clear insights into the mechanisms of the DSF-degrading activity of bacteria. Hence, a whole-genome analysis was performed to decipher the complete set of genes involved in DSF degradation. The QL-1 strain with the accession number MF988365.1 was provided by the Integrative Microbiology Research Centre, South China Agricultural University, China, and was isolated from agricultural soil [21]. Strain QL-1 was revived from storage at −80 °C by inoculating the culture substrate on the LB medium for 1 day. Genomic DNA was extracted with the sodium dodecyl sulfate (SDS) method. The harvested DNA was detected by agarose gel electrophoresis and quantified by Qubit. The genome of *A. lactucae* QL-1 was sequenced by Single Molecule, Real-Time (SMRT) technology. Sequencing was performed by LC-Bio Technology Co., Ltd., Hang Zhou, Zhejiang Province, China. The low-quality reads were filtered by the SMRT Link v5.0.1 (https://www.pacb.com/support/software-downloads/), and the filtered reads were assembled to generate one contig without gaps [51,52]. The original subordinate data is saved in the H5 format file, which contains sequencing sequence, base mass value, and other information. By controlling the quality of the original subordinate data and removing the low-quality sequence, the clean reads which can be used for analysis are obtained. Clean reads were counted, and the total amount of data, reads length, quality value distribution, and other information were obtained. Reads were assembled with SMRT Link V5.0.1 software, and further polished using the Illumina short-read sequencing data. (1) PacBio Sequel platform: Libraries for single-molecule real-time (SMRT) sequencing was constructed with an insert size of 10 kb using the SMRT bell TM Template kit, version 1.0. Briefly, the entire process had the following six steps: (i) Fragmentation and concentration of DNA; (ii) repairment of damaged DNA ends; (iii) preparation of blunt ligation reaction; (iv) purification of SMRTbell Templates with 0.45XAMPure PB Beads; (v) size-selection using the BluePippin System; (vi) repair of DNA damage after size-selection. Finally, the library quality was assessed on the Qubit^®^ 2.0 Fluorometer (Thermo Scientific, Waltham, MA, USA) and detected the insert fragment size by Agilent 2100 (Agilent Technologies). (2) Illumina NovaSeq platform: A total amount of 1 μg DNA per sample was used as input material for the DNA sample preparations. Sequencing libraries were generated using NEBNext^®^ Ultra™ DNA Library Prep Kit for Illumina (NEB, Ipswich, MA, USA) following the manufacturer’s recommendations, and index codes were added to attribute sequences to each sample. Briefly, the DNA sample was fragmented by sonication to a size of 350 bp, then DNA fragments were end-polished, A-tailed, and ligated with the full-length adaptor for Illumina sequencing with further PCR amplification. Finally, PCR products were purified (AMPure XP system, Beckman Coulter, Beverly, CA, USA), and libraries were analyzed for size distribution by Agilent 2100 Bioanalyzer and quantified using real-time PCR. The completeness of the genome assembly was evaluated by using BUSCO v4.1.2 (http://busco.ezlab.org) with the Pseudomonadales_odb10 benchmarking data set.

### 4.3. Genome Component Prediction and Gene Function

After obtaining the whole-genome data of *A. lactucae* QL-1, genes were used for genome component prediction, including the prediction of the coding gene, repetitive sequences, non-coding RNA, genomic islands, transposons, prophages, and clustered regularly interspaced short palindromic repeat sequences (CRISPR). The available steps were conducted as follows: (1) For bacteria, the related coding gene was retrieved using the GeneMarkS program (GeneMark, Atlanta, GA, USA); (2) the dispersed repetitive sequences (DRs) were predicted by the RepeatMasker (Version open-4.0.5) (http://www.repeatmasker.org/), and the tandem repeat sequences (TRs) were analyzed by the Tandem Repeats Finder, Version 4.07b (TRF) [53,54]; (3) analyses of non-coding RNAs, such as Transfer RNA (tRNA) gene prediction analyses, were carried out using the tRNAscan-SE (Version 1.3.1), and Ribosome RNA (rRNA) genes were identified by the rRNAmmer (Version 1.2) [55,56]. Small nuclear RNAs (snRNA) were predicted by BLAST against the Rfam database [57,58]; (4) the genomic islands were predicted using the IslandPath-DIOMB (Version 0.2) program (https://github.com/brinkmanlab/islandpath), and the transposons were predicted by transposon PSI based on the homologous blast method [59,60]. The PHAST (Version 2.3) was used for prophage prediction (http://phast.wishartlab.com/), and the CRISPR Finder (Version 1.0) was used for Clustered Regularly Interspaced Short Palindromic Repeat Sequences (CRISPR) identification [61,62].

Several databases, including respective Gene Ontology (GO) [63], the Kyoto Encyclopedia of Genes and Genomes (KEGG) [64,65], Clusters of Orthologous Groups (COG) [66], the Non-Redundant Protein Database (NR) [67], the Transporter Classification Database (TCDB) [68], protein families (Pfam), and Swiss-Prot [69], were used to predict gene functions. A whole-genome BLAST search (E-value less than 1 × 10^−5^ minimal alignment length percentage larger than 40%) was performed against the above databases, and the genes were annotated by aligning them with genes deposited in these databases. The prediction of Carbohydrate-Active enzymes was carried out based on the Carbohydrate-Active enzymes (CAZy) database [70].

The prediction of secretory proteins and Type I-VII proteins secreted by the pathogenic bacteria was based on the SignalP [71]. Meanwhile, the secondary metabolism gene clusters were analyzed by antiSMASH (version 2.0.2) (http://antismash.secondarymetabolites.org) [72]. In addition to the above, analyses of the pathogenicity and drug resistance were performed by Pathogen Host Interactions (PHI) [73], Virulence Factors of Pathogenic Bacteria (VFDB) [50], and the Antibiotic Resistance Genes Database (ARDB) [74], respectively. Furthermore, the Circos software was used to graph the circular genome data visualization [75].

### 4.4. Genome-Based Taxonomic Classification Analysis

The genome sequence of QL-1 was uploaded to the Type (Strain) Genome Server (TYGS), a free bioinformatics platform available under https://tygs.dsmz.de, for a whole genome-based taxonomic analysis [76]. The results were provided by the TYGS on 02 February 2020. Determination of closest type strain genomes was done in two complementary ways: First, all user genomes were compared against all type strain genomes available in the TYGS database via the MASH algorithm, a fast approximation of intergenomic relatedness [77], and the ten type strains with the smallest MASH distances were chosen per user genome. Second, an additional set of ten closely related type strains was determined via the 16S rDNA gene sequences. These were extracted from the user genomes using RNAmmer [56], and each sequence was subsequently BLASTed [78] against the 16S rDNA gene sequence of each of the currently 12,146 type strains available in the TYGS database. This was used as a proxy to find the best 50 matching type strains (according to the bitscore) for each user genome and to subsequently calculate precise distances using the Genome BLAST Distance Phylogeny approach (GBDP) under the algorithm ‘coverage’ and distance formula d5 [79]. These distances were finally used to determine the 10 closest type strain genomes for each of the user genomes. All pairwise comparisons among the set of genomes were conducted using GBDP and accurate intergenomic distances inferred under the algorithm ‘trimming’ and distance formula d5. One hundred distance replicates were calculated each. Digital DDH values and confidence intervals were calculated using the recommended settings of the GGDC 2.1. The resulting intergenomic distances were used to infer a balanced minimum evolution tree with branch support via FASTME 2.1.4, including SPR postprocessing [80]. Branch support was inferred from 100 pseudo-bootstrap replicates each. The trees were rooted at the midpoint and visualized with PhyD3 [81]. The type-based species clustering using a 70% dDDH radius around each of the 12 type strains was done as previously described [76].

### 4.5. Identification of the Gene Responsible for the Inactivation of DSF

All predicted protein sequences of *A. lactucae* QL-1 (obtained in this study) and *Xcc* ATCC 33,913 (downloaded from the NCBI RefSeq database) were analyzed by Inparanoid v4.1 to identify orthologs between the two bacteria. The similarity between *fadY* and *rpfB* was subsequently measured by pairwise BLASTP comparison between the two sequences [23].

### 4.6. Construction of an in-Frame Deletion Mutant and Complementation

To generate a *fadY* deletion mutant, 500 bp upstream and 500 bp downstream of the *fadY* gene were amplified by PCR with DNA-polymerase using the primer pairs De3487upF/De3487upR and De3487dnF/De3487dnR (Table 4). After purification with the Quick PCR purification kit, the PCR product was inserted into pK18mobsacB—a suicide vector—using the *BamHI* and *HindIII* sites for digestion by the homologous recombinase. The resultant construct was transformed into *E. coli* DH5*α*, individually, and then mobilized into strain QL-1 by triparental mating using the helper strain HB101 (RK2013) on LB plates at 30 °C for 10 h. The cells were suspended in sterile water, and appropriate dilutions were spread on LB plates containing chloramphenicol (to select against the donor strain) plus gentamicin (to select for a recipient with a non-replicating plasmid integrated into its chromosome). Several colonies were transferred to LB medium incubated overnight at 30 °C, and the cells were then suspended in sterile water and spread on LB plates containing 10% sucrose by appropriate dilutions. The generation of the ∆*fadY* deletion mutant was confirmed by PCR using external primer pair Ts3487-F/Ts3487-R and DNA sequencing analysis. The *fadY* open reading frame containing the 1680-nucleotide (nt) coding sequence of the QL-1 genome was deleted to generate the ∆*fadY* deletion mutant.

For complementation analysis, the coding region of *fadY*, together with its native promoter, was amplified by PCR using the specific primers listed in Table 4, and the PCR product was inserted into the HindHIII/BamHI sites of pBBR1MCS-5, resulting in the plasmid pBBR1MCS-5-*fadY*. The resultant construct was transformed into *E. coli* DH5α and mobilized into mutants by triparental conjugal mating. The complemented strains were confirmed by PCR analysis and DNA sequencing.

### 4.7. Expression and Purification of the FadY Protein

The GST-*fadY* fusion protein was purified as described in previous research [82]. The DNA fragment encoding *fadY* was amplified with the primers listed in Table 3 and subcloned into the expression vector pGEX-6p-1. Bacteria were grown at 37 °C in LB medium supplemented with 100 mg/mL ampicillin in shaking flasks, and isopropyl-b-D-thiogalactopyranoside (1 mM) was added to the bacterial culture when the OD_600_ reached about 0.6. After overnight culture at 18 °C, the cell pellet was re-suspended in phosphate-buffered saline (PBS) lysis buffer. The cells were then lysed by sonication, and the cell debris was removed by centrifugation at 12,000 rpm for 60 min and filtered through a 0.45 μm millipore filter (Millipore). The supernatants were loaded onto an affinity column containing glutathione-Sepharose 4F beads for affinity binding by the AKTA purification system, followed by washing with PBS lysis buffer and eluted 10 mM reduced glutathione. The purity of recombinant *fadY* was confirmed by SDS-PAGE analysis.

### 4.8. Codon Optimization of FadY

An amino acid that has two or more codes is called codon degeneracy; however, some of these codons expressed protein more easily than others. Based on that, the wild-type FadY amino acid sequence was sent to Genewiz Co., Ltd., Su Zhou, Jiangsu Province, China, to optimize codons, and higher purity FadY protein was obtained using codon degeneracy. The sequence comparison of wild-type and codon-optimized *fadY* genes is shown in Figure S1. An optimized DNA fragment was cloned into plasmid PGEX-6p-1 to express purity protein using the above method.

### 4.9. Determination of FadY Activity In Vitro

As previously described in several research studies, Ellman’s reagent was used to determine fatty acyl-CoA synthetase FadY activity by detecting the amount of free thiol (i.e., CoASH used in the reaction) in this study [83,84,85,86]. If FadY is a long-chain acyl-CoA synthetase, it can convert free fatty acids (FFA), and COASH can be converted into long-chain acyl-COA (Equation (1))

(1) $$\mathrm{ATP}\;+\;\mathrm{FFA}\;+\;\mathrm{COASH}\overset{\mathrm{FadY}}{\to }\mathrm{acyl}-\mathrm{COA}\;+\;\mathrm{AMP}\;+\;\mathrm{PPi}$$

As previously described by Kang et al. [87], reactions (450 mL total) were prepared with 100 μg of purified FadY in a reaction buffer which contained final concentrations of 150 mM Tris-HCl (pH 7.2), 10 mM MgCl_2_, 5 mM coenzyme A (CoASH), 5 mM ATP, 2 mM EDTA, 2 mM fatty acid (FA), and 0.1% Triton X-100 in 1.5 mL sterile centrifuge tubes. Recapitulating briefly, to start the reaction, all components described above (excluding CoASH) were mixed (405 mL total), and the mixture was pre-incubated at 37 °C for 3 min. Additionally, 5 mM CoASH was also pre-incubated at 37 °C for 3 min, at the same time. Then, the mixture was quickly mixed with the addition of 5 mM CoASH (45 mL), the reaction was initiated, and the sample was incubated at 37 °C during the course of the reaction. Immediately after mixing, a time zero point was taken by removing 75 mL from the 450 mL reaction mixture and adding it to 600 mL of 0.4 mM 5,5′-dithiobis-(2-nitrobenzoic acid) (DTNB, dissolved in 0.5 mg/mL EDTA which dissolved in 100 mM sodium hydrogen phosphate at pH 8.0) and the absorbance was measured at 412 nm. Subsequently, 75 mL aliquots of the reaction were taken at 0, 3, 5, 10, 20, and 30 min, respectively, and mixed with DTNB for additional measurements. Moreover, to verify the stability of CoASH and DTNB under the conditions listed above, control experiments with FadY enzymes treated with water bath heating for 5 min were performed to detect whether the absorbance changed at 412 nm. All of the reactions consisted of three replicates, and the experiments were repeated three times.

### 4.10. Virulence Tests

Experiments were carried out in a greenhouse to evaluate the suppressive effect of the gene *fadY* on black rot disease caused by *Xcc* in Chinese cabbage and radish. Healthy Chinese cabbage and radish seeds were planted and acclimatized in healthy soil under intermittent irrigation for 30 days, and the plants’ leaves were then excised with different bacterial treatments. Symptoms were recorded daily, and the plants were harvested when they grew for 30 days. The plants were grown in the greenhouse with a transparent screen to block out the rain. During the experimental period, the day/night temperature, photoperiod, and humidity were the same as the surrounding natural environment. The experimental design included three treatments, as follows: (1) Chinese cabbage and radish treated with distilled water and used as the control; (2) Chinese cabbage and radish treated with *Xcc* at 6 × 10^8^ CFU·mL^−1^; and (3) Chinese cabbage and radish treated with *Xcc* (*fadY*) at 6 × 10^8^ CFU·mL^−1^. All of the treatments consisted of three replicates, and the experiments were repeated three times [88].

### 4.11. Nucleotide Accession Number

The accession number for 16S rDNA gene sequences of the *A. lactucae* strain QL-1 deposited in the NCBI nucleotide sequence database is MF988365.1. The whole-genome sequence of QL-1 and sequence of FadY were also deposited in the NCBI database with accession numbers SUB7397134 and MT437357, respectively.

## 5. Conclusions

The present study first reported the genomic characteristics and DSF degradation mechanism of *A. lactucae* strain QL-1 isolated from agricultural soil. The results demonstrated that strain QL-1 was different from the type strain *A. lactucae* NRRL B-41902 and another DSF-degrading strain *Pseudomonas aeruginosa* PAO1. KEGG analysis suggested that the mapping pathways of genes were related to fatty acid degradation and metabolism. DSF degradation experiments in wild-type strain QL-1 and the *fadY* gene-deficient mutant proved that the gene *fadY* was necessary for the DSF degradation of strain QL-1. Additionally, the determination of FadY activity in vitro revealed that the fatty acyl-CoA synthetase FadY has excellent catalytic activity. Moreover, *Xcc*-expressing enzyme FadY lost virulence on host plants, indicating that FadY was capable of preventing and controlling black rot disease caused by *Xcc*. This study provides new insights into the QQ mechanisms of bacterial strain QL-1, and presents useful enzymes and related genes for the biocontrol of infectious diseases caused by DSF-dependent bacterial pathogens.

## Figures and Tables

**Table 1:** Genome characteristics of QL-1.

Genome Characteristics	
Total length (bp)	3,973,648
GC content (%)	40.04
Number of protein-coding genes	3.707
Average length of protein-coding genes (bp)	940
% of Genome (protein-coding genes)	87.71
rRNA genes	18
tRNA genes	73
Repeats	153
% of Genome (repeats)	0.37

**Table 2:** The change of absorbance in the identification of FadY.

Time (min)	FadY + FA			Control		
	1	2	3	1	2	3
0	0.565	0.586	0.578	0.934	0.887	0.936
3	0.452	0.426	0.415	0.912	0.905	0.900
5	0.382	0.377	0.334	0.943	0.922	0.899
10	0.221	0.233	0.221	0.878	0.925	0.910
20	0.128	0.162	0.115	0.904	0.921	0.890
30	0.000	0.000	0.000	0.869	0.869	0.824

**Table 3:** The list of strains and plasmids used in this study.

Strains or Plasmids	Relevant Genotype or Phenotype	Sources
**QL-1 strains**		
QL-1	Laboratory storage	This study
∆*fadY*	*fadY* deletion mutant of strain QL-1 with 1680-nt internal coding region deleted	This study
*Xcc* XC1	Pathogenic bacteria causing black rot	Lab collection
***Escherichia coli* strains**		
DH5*α*	*spuE44* ∆*lacU169(φ80lacZ*∆*M15) hsdR17λpir recA1 endA1 gyrA96 thi-1 relA1*	Lab collection
BL21	F^-^*ompThsdS* (r_B_^−^m_B_^−^) *dcm*^+^ Tet^r^ *gal* (DE3) *endA*	Lab collection
pRK2013	Tra^+^, Mob^-^, ColE1-replicon, Kan^r^, Spe^r^	Lab collection
**Plasmids**		
pBBR1-MCS5	Broad host-range cloning vector; Gm^r^	Lab collection
pK18mobsacB	Broad-host-range gene replacement vector, sacB,Gm^r^	Lab collection
pBBR1-MCS5-*fadY*	pBBR1-MCS5 containing *fadY* under control of P_lac_	This study
pK18-*fadY*	pK18mobsacB contain flanking of *fadY*	This study
pGEX-6p-1	GST fusion protein expression vector, Amp^r^	Invitrogen
pGEX-*fadY*	pGEX-6p-1 containing *fadY*	This study

Superscript “r” means “resistance”.

**Table 4:** Primers used in this study.

Primers	Sequence (5′–3′)	Applications
De3487upF	GAGCTCGGTACCCGGGGATCCGGAGCGCCTGGCGATCAT	For amplification of the 5′-region of *fadY*
De3487upR	CGGAGATAATGGCATTTAAGTTAATAAAAAAGCGCCTTAGGG	
De3487dnF	CTTAAATGCCATTATCTCCGATTCGT	For amplification of the 3′-region of *fadY*
De3487dnR	CGACGGCCAGTGCCAAGCTTAGTTGATACAACTTGAAGCG	
Ts3487-F	CTGCATAGTGCCATCCATCAC	For identification of ∆*fadY*
Ts3487-R	GCCAAGGCAGGAAAAAGC	
c*fadY*-F	GTCGACGGTATCGATAAGCTTAAGCTAGCGTCGGGCAACA	For construction of pBBR1-MCS5-*fadY*
c*fadY*-R	CGCTCTAGAACTAGTGGATCCAAATATAGAAACAAAAAAAGCGCCC	
p*fadY*-F	CAGTCAGTCACGATGCGGCCGATGGAAAAGATTTGGTTTGCAGA	For construction of pGEX-6P-1-*fadY*
p*fadY*-R	CCCCTGGGATCCCCGGAATTCTTAGGTTGGTTTACGTAAGTCTTTACG	

## Supplementary Materials

Supplementary Materials can be found at https://www.mdpi.com/1422-0067/21/18/6729/s1.
