# Supplementary material for: Whole-Genome Sequencing Analysis of Quorum Quenching Bacterial Strain Acinetobacter lactucae QL-1 Identifies the FadY Enzyme for Degradation of the Diffusible Signal Factor
Source: Int J Mol Sci. 2020 Sep 14;21(18):6729. doi: 10.3390/ijms21186729 (PMC7554724; doi:10.3390/ijms21186729)
Supplement: Supplementary file 1 [file ijms-21-06729-s001.pdf]

Supplementary materials

Whole-Genome Sequencing Analysis of Quorum Quenching Bacterial Strain *Acinetobacter lactucae* QL-1 Identifies the FadY Enzyme for Degradation of the Diffusible Signal Factor

Tian Ye[1,2#] , Tian Zhou[1,2#] , Xudan Xu[1,2#], Wenping Zhang[1,2], Xinghui Fan[1,2], Sandhya Mishra[1,2], Lianhui Zhang[1,2], Xiaofan Zhou[1,2*], Shaohua Chen[1,2*]

1 State Key Laboratory for Conservation and Utilization of Subtropical Agro-bioresources, Guangdong Province Key Laboratory of Microbial Signals and Disease Control, Integrative Microbiology Research Centre, South China Agricultural University, Guangzhou 510642, China

2 Guangdong Laboratory for Lingnan Modern Agriculture, Guangzhou 510642, China

* Correspondence: shchen@scau.edu.cn; xiaofan_zhou@scau.edu.cn; Tel+86-20-8528 8229

# These authors contributed equally to this work.

3.1.1 Repeat sequences

The length distribution of strain QL-1 coding genes is shown in Figure S4. The repeat sequence statistics of *A. lactucae* QL-1 are shown in Table S1. The total length of repeat sequences was 14,887 bp, covering 0.3746% of the genomic length. Among the repeat elements, long interspersed nuclear elements (LINEs) accounted for 0.0546% and short interspersed nuclear elements (SINEs) for 0.0332% of the assembled genome. The total length of 87 long terminal repeated sequences (LTR) was 10,433 bp, covering 0.2626% of the genomic length.

3.1.2. Non-coding RNAs

The results of non-coding RNAs in the *A. lactucae* QL-1 genome are shown in Table S2. With regard to RNA, 73 tRNAs, 18 rRNAs, and 1 sRNA were predicted. Among the rRNAs, 6 5S_rRNAs, 6 16S_rRNAs, and 6 23S_rRNAs were obtained.

3.1.3. Functional annotation

A total of 3707 protein-encoding genes were verified using publicly available databases, including the GO, KEGG, COG, NR, Pfam, TCDB, Swiss-Prot, and CAZy protein databases. According to the GO analysis of *A. lactucae* QL-1, 2544 predicted proteins accounting for 33.07% of the entire genome were identified; they were divided into three major subclasses: Molecular function (10 branches), cellular component (11 branches), and biological process (24 branches). These were mainly distributed across four functional entries, including "Cellular process", "Metabolic process", "Binding", and "Catalytic activity", for which the numbers of annotated genes were 1370, 1453, 1113, and 1315, respectively (Figure S5).

The KEGG function classification is shown in Figure S6. To further understand the gene functions in *A. lactucae* QL-1, 3586 putative proteins (accounting for 96.74% of the total number of genes) were successfully assigned to their orthologues in the KEGG database enriched in 217 metabolic pathways (KEGG pathways). "Metabolic pathways" (ko01100) had the highest number of genes (593). This was followed by "Biosynthesis of secondary metabolites" (ko01110) (249), "Microbial metabolism in diverse environments" (ko01120) (239), "Biosynthesis of antibiotics" (ko01130) (192), and "Biosynthesis of amino acids" (ko01230) (112), which were the most gene-rich classes in the KEGG pathway groupings. Notably, it was found that 26 genes in *A. lactucae* QL-1 were involved in fatty acid degradation (ko00071), which further confirmed that QL-1 is capable of the enzymatic inactivation of DSF.

NCBI COG mapping revealed that 3299 proteins were assigned to COG categories, accounting for 88.99% of the total number of coding genes. "General function prediction only" had the highest number of genes (334), which were not unambiguously assigned to a particular group. This was followed by "Amino acid transport and metabolism" (299), "Transcription" (292), "Lipid transport and metabolism" (259), "Translation, ribosomal structure and biogenesis" (225), and "Energy production and conversion" (215), which were the most gene-rich classes in the COG groupings (Figure S7).

Among the protein-coding genes, 3637 genes were annotated in the NR database, accounting for 98.11% of the total number of genes, and 1642 genes were annotated in the SwissProt database, accounting for 44.29% of the total number of genes.

The Venn map of *A. lactucae* QL-1 was obtained according to the annotation results of the COG, NR, Swissprot, and KEGG protein predicted genes, and is shown in Figure S8. It showed 1563 mutual genes, accounting for 42.16% of the total protein-predicted genes. In addition, 55, 2, 0, and 0 specific genes were found in the NR, KEGG, KOG, and Swissport databases, respectively.

The gene annotation information indicated that there were 3640, 3586, 2885, 2544, 1642, 394, 261, and 86 annotated genes in the NR, KEGG, COG, GO, Swiss-Prot, TCDB, PHI, and CAZy protein data.

3.1.4. Carbohydrate-active enzyme (CAZyme)

The genomes were mapped with the CAZy database to study the presence of CAZymes. A total of 91 genes could be assigned to CAZymes families, as defined in the CAZy database (Figure S9). Glycosyl Transferases (GTs) had the highest number of genes (34), followed by Glycoside Hydrolases (GHs) (30), Carbohydrate-Binding Modules (CBMs) (18), Carbohydrate Esterases (CEs) (6), Auxiliary Activities (AA) (2), and Polysaccharide Lyases (PL) (1).

3.1.5. Gene clusters in secondary metabolite prediction

The gene clusters involved in the secondary metabolism of *A. lactucae* QL-1 are shown in Table S3. Six gene clusters, including 2 arylpolyene, 1 bacteriocin, 1 nrps, 1 siderophorerps, and 1 hserlactone-nrps, were predicted in the *A. lactucae* QL-1 genome.

3.1.6. The pathogen–host interaction (PHI) annotations

The PHI is a database of pathogen–host interactions, mainly derived from fungi, oocytes, and bacterial pathogens which are capable of infecting hosts, including animals, plants, fungi, and insects. This database also includes antifungal compounds and their corresponding target genes, which is important in finding target genes for drug interventions. Each gene in the database contains sequences of nucleic acids and amino acids, as well as a detailed description of the function of the proteins predicted during infection with the host. The number of pathogenicity-related genes (261) in the PHI database of QL-1 is shown in Figure S10. Among these, there were 3 chemistry target genes (resistance to chemicals), 2 effector-related genes (plant avirulence determinant), 21 increased virulence (hypervirulence)-related genes, 9 lethal genes, 12 genes related to a loss of pathogenicity, 157 genes related to reduced virulence, and 57 genes related to unaffected pathogenicity.

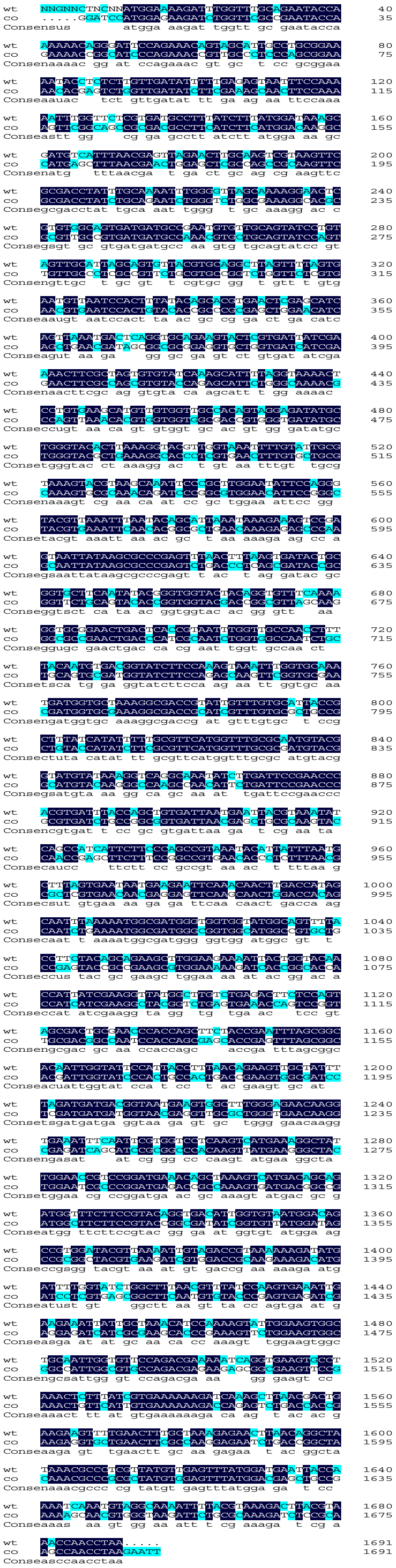

Figure 1. Sequence comparison of wild-type and codon-optimized *fadY* genes. wt: wild-type *fadY* genes; co: codon-optimized *fadY* genes.

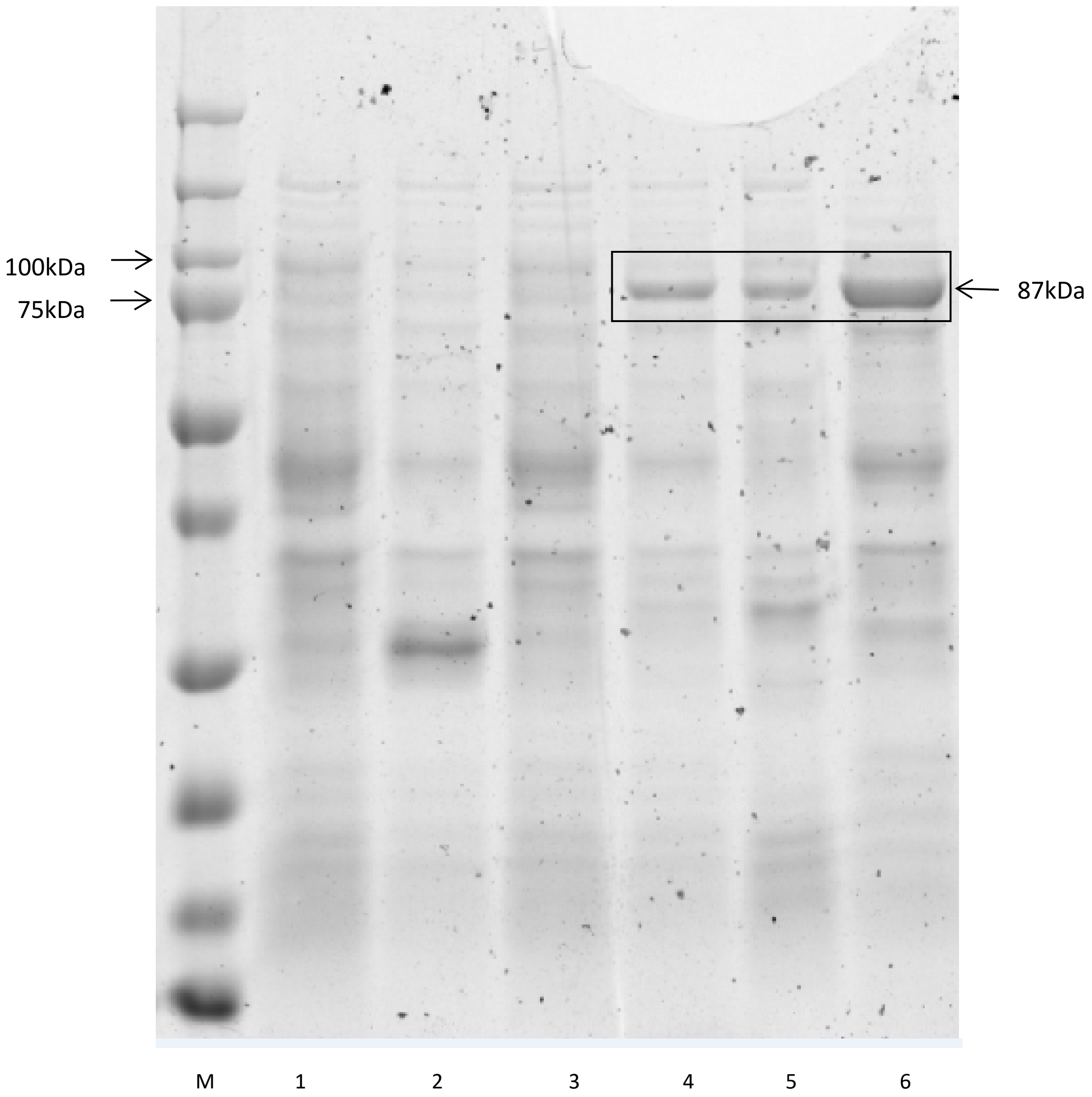

Figure 2. Expression and purification of enzyme FadY. M: marker; 1: total protein of BL21 harboring pGEX-6p-1; 2: total protein BL21 harboring pGEX-6p-1 with IPTG; 3: total protein of BL21 harboring recombinant pGEX-6p-1-fadY; 4: total protein of BL21 harboring recombinant pGEX-6p-1-*fadY* with IPTG; 5: pellet protein of BL21 harboring recombinant pGEX-6p-1-*fadY* with IPTG; 6: supernatant protein of BL21 harboring recombinant pGEX-6p-1-*fadY* with IPTG.

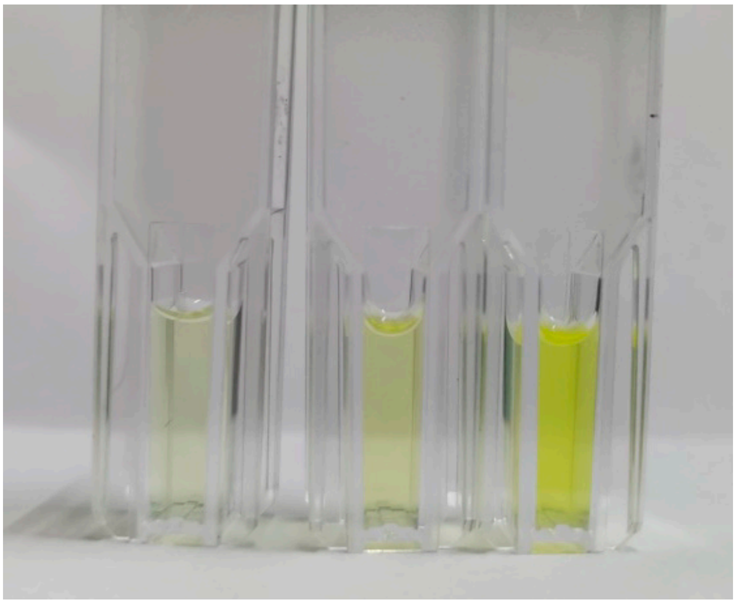

Figure 3. From left to right are 5,5'-dithiobis-(2-nitrobenzoic acid) (DTNB), sample mixed with DTNB, and control mixed with DTNB, respectively.

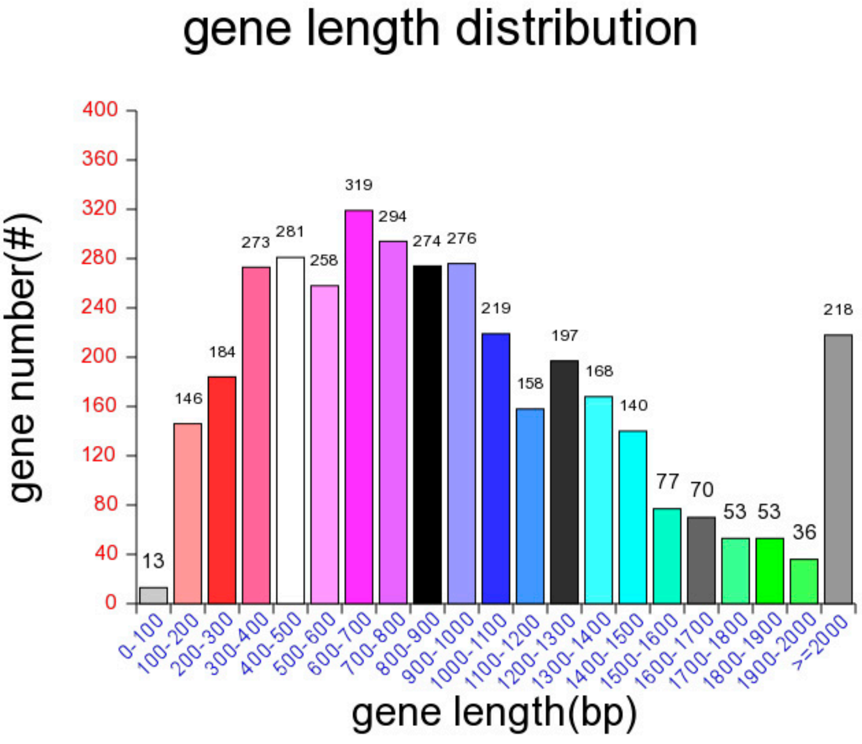

Figure 4. The gene length distribution of *Acinetobacter lactucae* QL-1 coding genes.

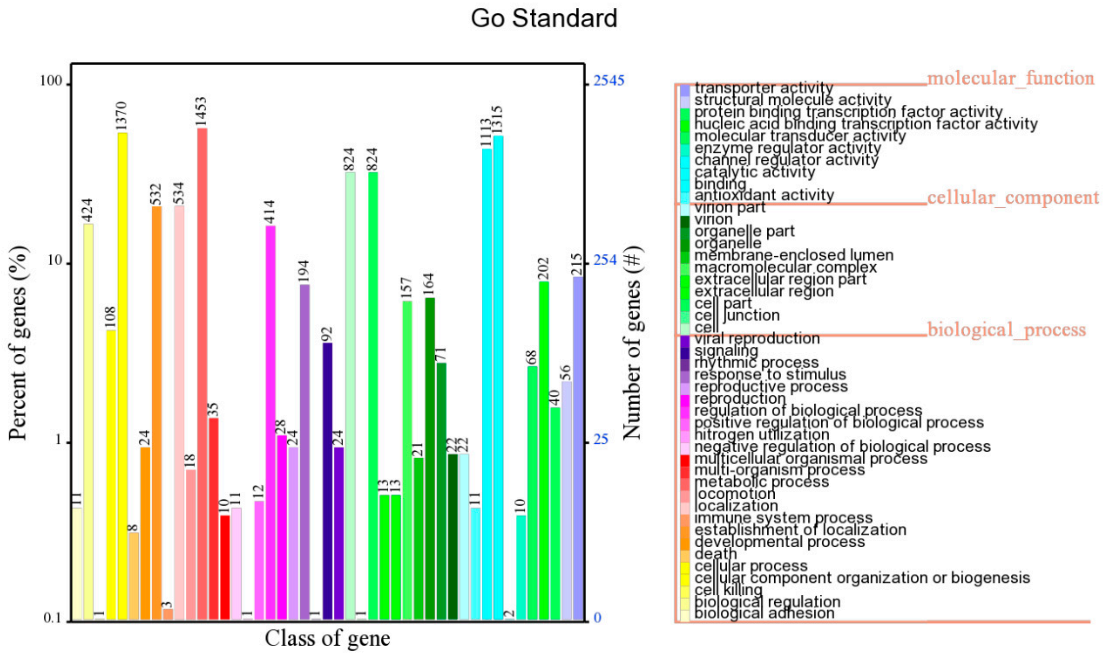

Figure 5. Gene Ontology (GO) functional annotation of *Acinetobacter lactucae* QL-1.

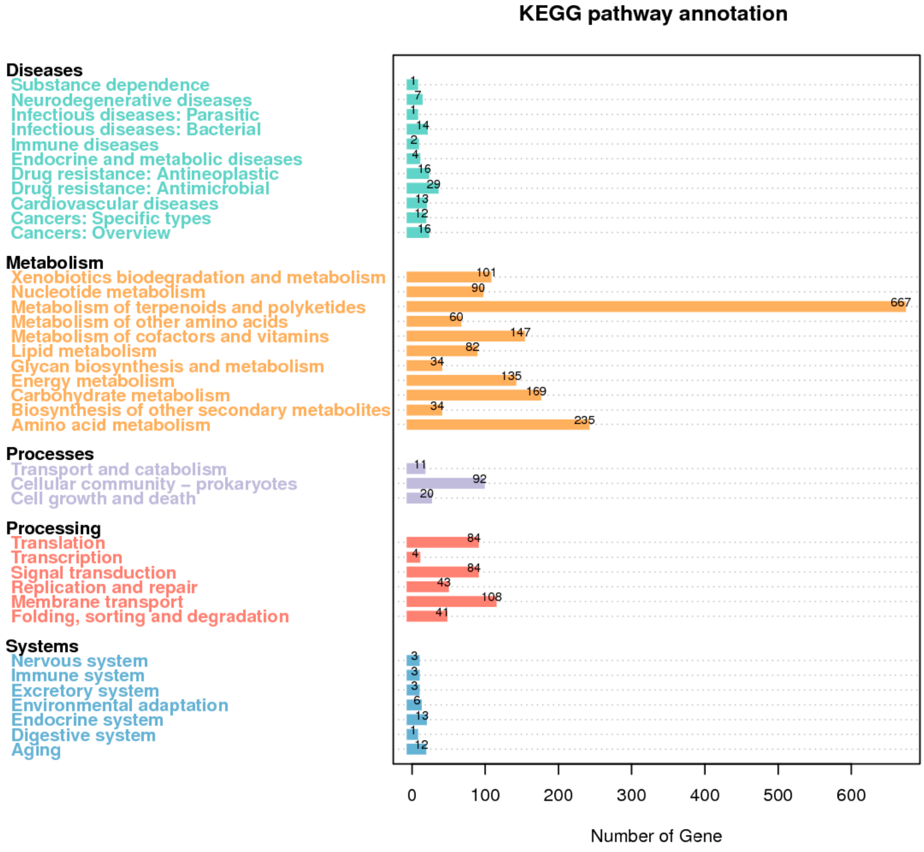

Figure 6. The Kyoto Encyclopedia of Genes and Genomes (KEGG) function annotation of *Acinetobacter lactucae* QL-1.

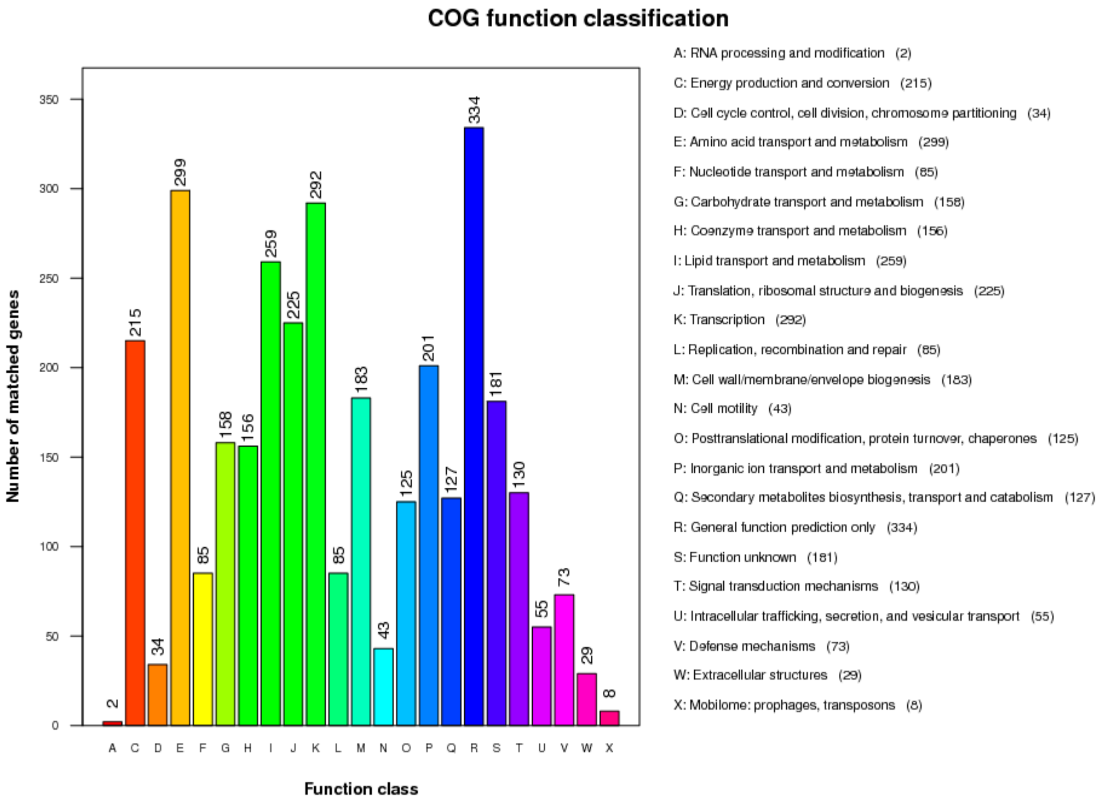

Figure 7. Clusters of orthologous groups of proteins (COG) function classification of proteins in *Acinetobacter lactucae* QL-1.

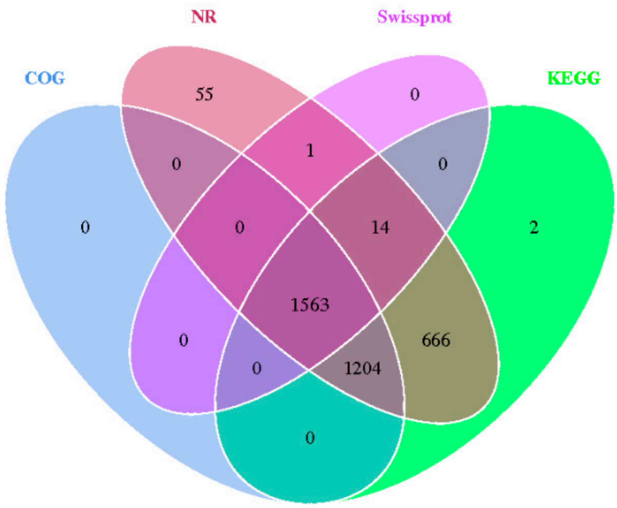

Figure 8. Venn diagrams of the COG, Non-Redundant Protein Database (NR), Swissport, and KEGG annotated genes.

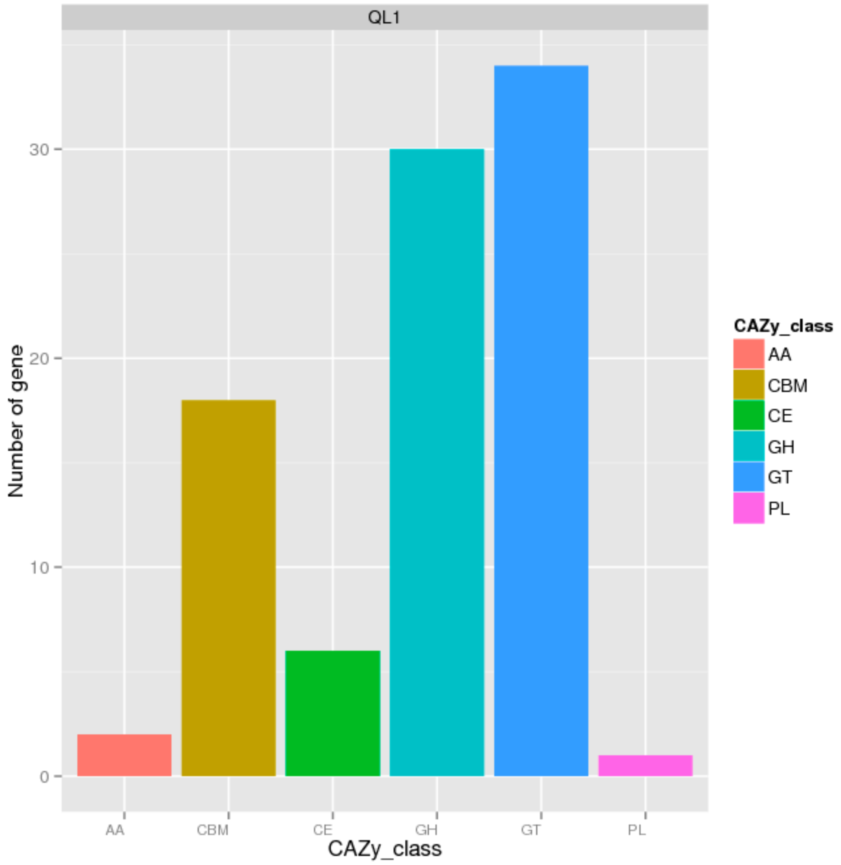

Figure 9. The carbohydrate-active enzyme (CAZyme) annotations of *Acinetobacter lactucae* QL-1.

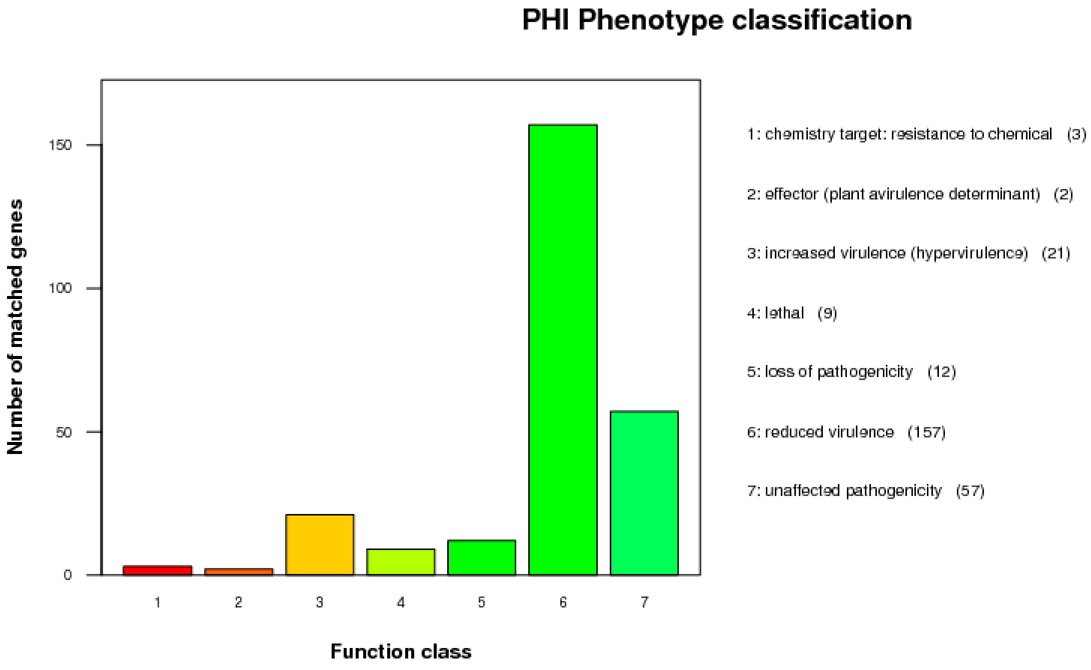

Figure 10. The pathogen–host interaction (PHI) annotations of *Acinetobacter lactucae* QL-1.

Table 1. Statistical results of repeat sequences in the strain QL-1 genome.

Type	Number of elements	Total length (bp)	Percentage of genome (%)	Average length (bp)
LTR	87	10,433	0.2626	120
DNA	16	1,167	0.0294	73
LINE	29	2,168	0.0546	75
SINE	20	1,321	0.0332	66
RC	0	0	0	0
Unknown	1	71	0.0018	71
Total	153	14,887	0.3746	99

Table 2. Statistical results of ncRNA in the strain QL-1 genome.

Type		Number	Average length (bp)	Total length (bp)	Percentage of genome (%)
tRNA		73	77	5,680	0.1429
	5s_rRNA	6	114	684	
rRNA_	16s_rRNA	6	1,526	9,156	0.6839
	23s_rRNA	6	2,889	17,334	
sRNA		1	89	89	0.0022

Table 3. The gene clusters involved in the secondary metabolism of *Acinetobacter lactucae* QL-1.

Clusters	Clusters number	Gene number
Bacteriocin	1	16
Arylpolyene	2	84
Nrps	1	51
Siderophore	1	14
Hserlactone-nrps	1	48
